# Recent advances in copper-catalyzed direct hydroamination of alkenes with (hetero)aromatic amines

**DOI:** 10.3762/bjoc.22.73

**Published:** 2026-06-11

**Authors:** Hyejeong Lee, Yunmi Lee

**Affiliations:** 1 College of Pharmacy, Duksung Women’s University, Seoul 01369, Republic of Korea; 2 Department of Chemistry, Kwangwoon University, Seoul 01897, Republic of Korea

**Keywords:** aromatic amines, aza-Michael addition, Cu catalysis, hydroamination, *N*-heterocycles

## Abstract

Nitrogen-containing aromatic amines and aza-heterocycles are ubiquitous motifs in pharmaceuticals and functional materials, making efficient C–N bond formation a fundamental transformation in synthetic chemistry. The direct hydroamination of alkenes using (hetero)aromatic N–H nucleophiles offers an atom- and step-economical strategy; however, the reduced nucleophilicity and distinct coordination behavior of these substrates render such transformations inherently challenging. Recently, copper catalysis has emerged as a versatile and practical platform for addressing these limitations. Owing to its redox flexibility and ligand tunability, copper facilitates multiple activation modes, including copper–amido-mediated nucleophilic addition or aminocupration, Lewis acid-type alkene activation, and radical-mediated pathways. These complementary mechanisms provide access to diverse alkene classes with control over regioselectivity (Markovnikov vs anti-Markovnikov addition), stereoselectivity, and functional group tolerance. This review summarizes recent advances in the copper-catalyzed hydroamination of alkenes with (hetero)aromatic N–H nucleophiles, emphasizing the mechanistic paradigms and the factors governing selectivity.

## Introduction

Nitrogen-containing aromatic compounds, particularly aromatic amines and aza-heterocycles, are indispensable structural motifs in pharmaceuticals, agrochemicals, natural products, and advanced functional materials [1–4]. Their unique electronic characteristics, tunable basicity, and directional hydrogen-bonding capabilities enable precise modulation of molecular recognition, biological activity, and physicochemical properties. Consequently, the efficient and selective construction of carbon–nitrogen (C–N) bonds embedded within aromatic nitrogen frameworks remains a primary objective of modern organic synthesis [5–6]. Among the diverse strategies available for C–N bond formation, the direct addition of an N–H bond across a C–C double bond, known as hydroamination, is an ideal transformation considering atom and step economy [7–12]. Hydroamination simultaneously forms C–N and C–H bonds from simple and readily available starting materials without prefunctionalization. When applied to electron-deficient alkenes such as α,β-unsaturated carbonyl compounds, nitroalkenes, or vinyl sulfones, this transformation is often categorized as an aza-Michael addition [13–18]. In such cases, the reaction provides direct access to β-amino carbonyl derivatives and related motifs that serve as versatile intermediates in synthetic and medicinal chemistry.

**Scheme 1:**
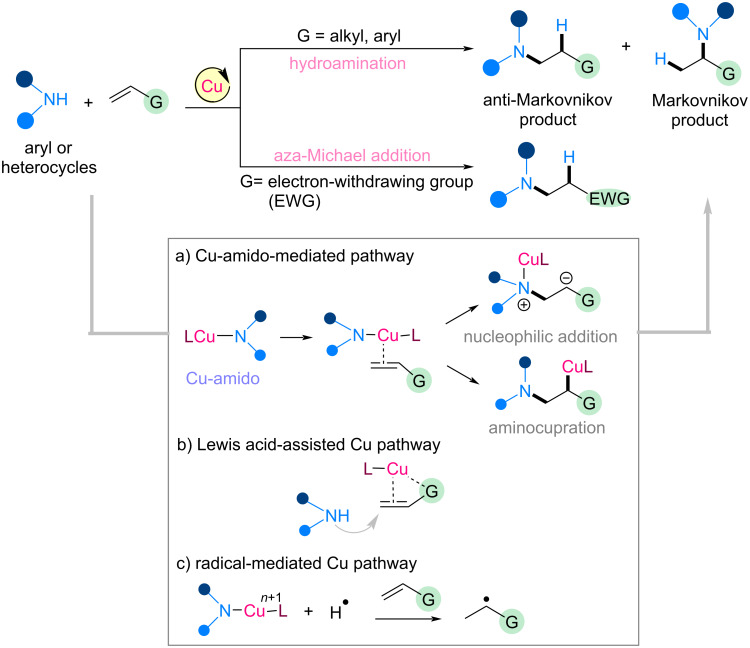
Mechanistic pathways in copper-catalyzed hydroamination.

Despite its conceptual simplicity, catalytic hydroamination remains challenging. This is because the direct interaction between an electron-rich nitrogen lone pair and a relatively inert alkene π-system is often kinetically disfavored in the absence of activation [19]. Furthermore, the hydroamination of unsymmetrical alkenes introduces additional complexity in controlling the regioselectivity (Markovnikov vs anti-Markovnikov addition), while stereochemical control (diastereo- and enantioselectivity) presents further challenges (Scheme 1) [7]. These challenges become more pronounced when using (hetero)aromatic amines as the N–H partners. Compared to aliphatic amines, aromatic amines and aza-heterocycles often exhibit attenuated nucleophilicity, distinct acidity profiles, and potential coordination to metal centers, all of which can significantly influence catalytic reactivity and selectivity [20]. Therefore, the development of catalytic systems that can accommodate the unique reactivities of (hetero)aromatic N–H nucleophiles remains challenging.

To address these issues, a range of transition metal catalysts have been explored [21–23], with copper emerging as a potent candidate [24–27]. Beyond its abundance, low cost, and comparatively low toxicity relative to precious metals such as palladium or rhodium, copper offers exceptional mechanistic versatility. The accessible Cu(I)/Cu(II)/Cu(III) redox manifold enables classical two-electron pathways and single-electron radical processes within broadly similar catalytic environments [28–29]. This flexibility is particularly valuable in hydroamination chemistry.

Several distinct yet interrelated activation modes have been identified for copper-catalyzed alkene amination [27]. Copper frequently participates directly in amine activation via the formation of Cu–N species (Scheme 1a) [30–31]. In the presence of a base and suitable ligands such as *N*-heterocyclic carbenes or electron-rich phosphines, the deprotonation of an N–H heteroaromatic can generate a copper–amido intermediate [32–34]. These species typically exhibit enhanced nucleophilicity and can engage alkenes via the direct nucleophilic addition of a copper–amido intermediate or aminocupration-type pathways, enabling efficient C–N bond formation under relatively mild conditions. Copper species may also operate in a Lewis acid-type capacity, where coordination to the alkene or copper-derived Brønsted acidity generated in situ increases alkene electrophilicity and lowers the barrier for nucleophilic attack (Scheme 1b). Further, copper can mediate radical-based pathways either by generating hydrogen radicals or intercepting nitrogen-centered radical intermediates in oxidative- or photoredox-assisted systems (Scheme 1c). These radical manifolds often provide complementary regioselectivity profiles and an expanded substrate scope compared to purely polar mechanisms.

The ability of copper catalysts to operate across these mechanistic regimes has enabled significant progress in the hydroamination of activated and challenging alkenes using (hetero)aromatic N–H nucleophiles. Notably, ligand design has emerged as a powerful tool for tuning the reactivity and selectivity, including control over Markovnikov vs anti-Markovnikov addition and enantioselective induction in selected cases. These developments collectively underscore the unique adaptability of Cu as a catalytic platform for C–N bond construction.

Unlike earlier review articles that broadly covered copper-catalyzed C–N bond-forming reactions or hydroamination chemistry across diverse nitrogen sources [24–27], this review places a clear and specific emphasis on recent advances in the direct hydroamination of alkenes using aromatic amines and aza-heterocyclic N–H nucleophiles under copper catalysis. The primary focus is on the mechanistic paradigms, Cu–amido pathways, Lewis acid activation, and carbon-radical manifolds, and on how these distinct activation modes translate into predictable control over regioselectivity, stereoselectivity, and functional group tolerance. By organizing the field according to the catalyst role and the selectivity-determining step, this review aims to provide a coherent framework for understanding the role of copper catalysis in shaping efficient and selective hydroamination chemistry.

## Review

### Copper-catalyzed aza-Michael addition

#### Early studies on copper–amido complexes by Gunnoe

The aza-Michael addition of (hetero)aromatic amines to electron-deficient olefins provides a straightforward and atom-economical route to β-amino carbonyl, β-amino sulfone, and β-amino nitrile motifs, which are commonly found in biologically active molecules. However, the relatively low nucleophilicity of aromatic amines and *N*-heteroaromatic substrates often limits their reactivity in conjugate addition reactions. To overcome this limitation, transition metal catalysis has been widely explored. Among the various catalytic systems, copper catalysts have attracted particular attention owing to their ability to activate amine components by forming copper–amido species. In this activation mode, deprotonation of the N–H bond generates a Cu–N intermediate with enhanced nucleophilicity, facilitating conjugate addition to electron-deficient alkenes. The formation of such well-defined copper–amido complexes provides valuable mechanistic insights into copper-catalyzed hydroamination and conjugate addition reactions.

**Scheme 2:**
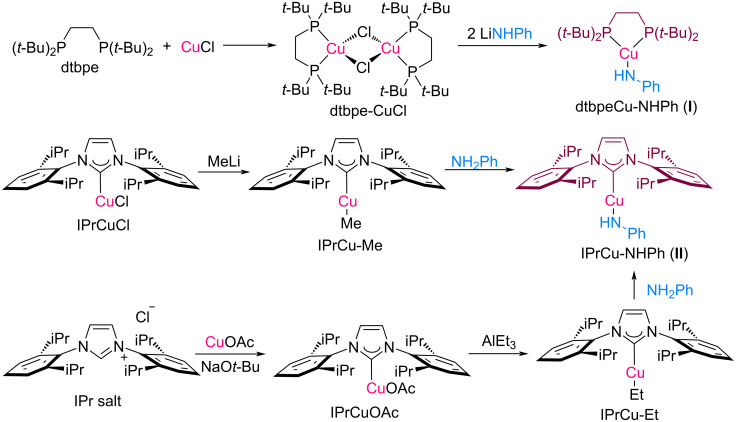
Synthesis of copper–amido complexes.

Pioneering studies by Gunnoe et al. describe the synthesis and characterization of well-defined copper–amido complexes **I** and **II**, supported by electron-donating ligands such as phosphines and *N*-heterocyclic carbenes (NHCs) (Scheme 2) [32–34]. Copper–amido complex **I** was prepared by treating the dtbpe-ligated Cu–Cl complex (dtbpe = 1,2-bis(di-*tert*-butylphosphino)ethane) with LiNHPh, while the NHC-based complex **II** was generated by the reaction of IPrCu-Me or IPrCu-Et (IPr = 1,3-bis(2,6-diisopropylphenyl)imidazol-2-ylidene) with aniline. These well-defined copper–amido complexes serve as important model systems for elucidating the role of Cu–N species in catalytic C–N bond formation. Structural and reactivity studies revealed that electron-rich ligands stabilized the copper center while facilitating the formation of copper–amido intermediates, consequently enhancing the nucleophilicity of the nitrogen atom.

**Scheme 3:**
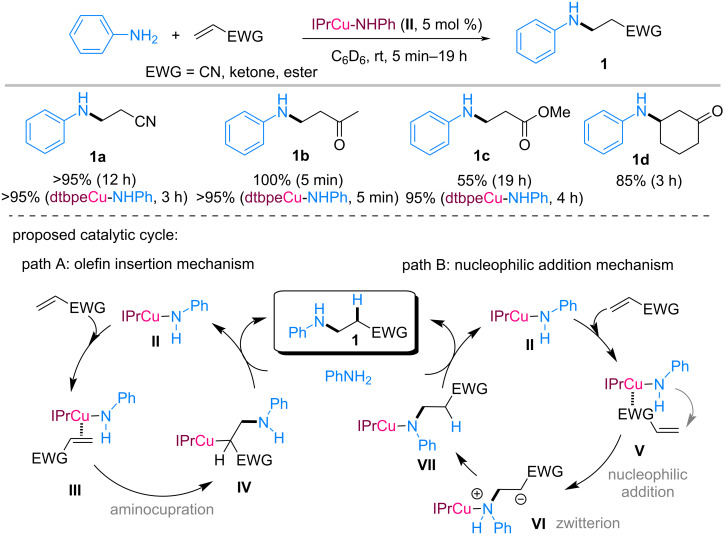
Cu–amido catalysts studied for the aza-Michael addition of aniline to electron-deficient olefins.

The catalytic activities of complexes **I** and **II** (Scheme 2) were subsequently evaluated by adding aniline to activated olefins (Scheme 3) [35–36]. Using 5 mol % of the copper–amido catalyst at room temperature, the α,β-unsaturated substrates including acrylonitrile, methyl vinyl ketone, methyl acrylate, and cyclic enones were converted to the corresponding β-amino products **1a**–**d** with excellent regioselectivity. These reactions proceeded exclusively in an anti-Markovnikov manner with high efficiency. For example, the addition of aniline to acrylonitrile afforded **1a** with >95% conversion after 12 h using IPrCu–NHPh (**II**), whereas methyl vinyl ketone was rapidly transformed to **1b**, reaching quantitative conversion within 5 min. For **1c**, the sterically demanding dtbpeCu-NHPh (**I**) exhibited comparable or enhanced activity, highlighting the significant influence of the ligand environment on catalytic performance. These initial studies were conducted in C_6_D_6_ solvent without isolating the products, and the substrate scope was not explored extensively.

Mechanistic investigations suggest two possible pathways for the copper-catalyzed aza-Michael addition of anilines to electron-deficient olefins (Scheme 3). In path A, the copper amido complex **II** initially coordinated to the activated olefin, followed by aminocupration across the C=C bond to form the alkylcopper intermediate **IV**, which subsequently underwent proton transfer with aniline to furnish the β-amino product **1** while regenerating the copper–amido species **II**. Alternatively, path B proceeded through the direct nucleophilic addition of copper–amido complex **II** to the electron-deficient olefin. In this pathway, the nucleophilic attack of the copper–amido species on the activated alkene generated zwitterionic intermediate **VI**, followed by proton transfer to afford product **1**. Control experiments revealed that simple copper salts or free ligands alone were ineffective, indicating that discrete copper–amido complexes served as catalytically active species, rather than merely functioning as Lewis acids. Although both pathways are mechanistically plausible, experimental evidence supports the nucleophilic addition pathway (path B). In particular, NMR analysis indicated the formation of the organocopper species (IPr)Cu(N(Ph)CH_2_CH_2_CN) (**VII**), a key intermediate consistent with the nucleophilic addition mechanism. These early mechanistic studies established that copper-bound amido species act as key nucleophilic intermediates in alkene amination, providing a mechanistic foundation for the subsequent development of copper-catalyzed aza-Michael and hydroamination reactions involving (hetero)aromatic amines.

#### Aza-Michael addition via copper–amido intermediate

**Scheme 4:**
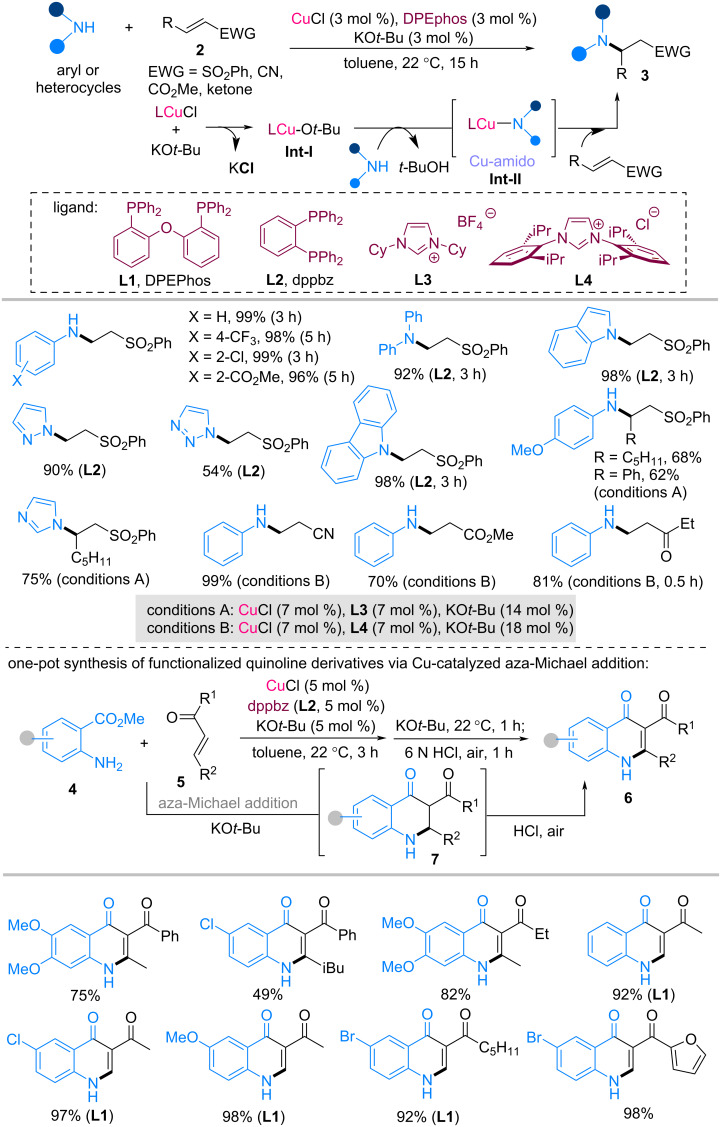
Copper-catalyzed aza-Michael addition of (hetero)aromatic amines to α,β-unsaturated alkenes.

Following the establishment of copper–amido platforms for aza-Michael reactions, the Lee group further expanded the scope of copper-catalyzed C–N bond formation using (hetero)arylamines with diverse α,β-unsaturated olefins and dienes. As shown in Scheme 4, a practical copper-catalyzed aza-Michael addition of (hetero)aromatic amines to α,β-unsaturated olefins **2** is developed using an inexpensive CuCl catalyst in combination with electron-donating phosphine ligands (**L1, L2**) or NHC salts (**L3, L4**) and KO*t*-Bu [37]. Various aromatic amines and heteroaromatic N–H nucleophiles reacted efficiently with electron-deficient alkenes **2**, such as vinyl sulfones, acrylonitrile derivatives, and α,β-unsaturated carbonyl compounds, to afford the corresponding β-amino products **3** in excellent yields (up to 99%). This method exhibited high functional group tolerance on aromatic amine substrates, including those with electron-donating and electron-withdrawing substituents, without significantly affecting the reaction efficiency. In addition to simple anilines, diverse heteroaromatic amines including indole, pyrrole, triazole, carbazole, and imidazole derivatives participated effectively in the copper-catalyzed conjugate addition, providing the corresponding β-amino products **3** in high yields.

Mechanistically, the transformation is proposed to proceed via a copper–amido catalytic cycle, which is analogous to the Gunnoe-type copper–amido nucleophilic addition or aminocupration pathway, as illustrated in Scheme 3. In this process, a phosphine- or NHC-ligated copper–amido species **Int-II** was generated in situ via the reaction of the ligand-bound Cu–O*t*-Bu intermediate **Int-I** with the amine substrate. In contrast to the NHC–CuNHPh catalyst system reported by the Gunnoe group, which requires the preparation of a preformed NHC–CuNHPh complex, the present protocol generates catalytically active copper–amido species directly in situ from CuCl, the ligand, and KO*t*-Bu. Consequently, the reaction can be performed under simple benchtop conditions without requiring glovebox techniques, providing a more operationally convenient and practical method. Furthermore, the synthetic utility of this methodology is demonstrated via a one-pot sequence consisting of copper-catalyzed aza-Michael addition followed by base-mediated cyclization and acid-promoted aerobic oxidation to afford quinolinone derivatives (Scheme 4) [38–39]. In this sequence, 2-aminobenzoate derivatives **4** reacted with α,β-unsaturated carbonyl compounds **5** to form the β-amino ketone intermediates, which subsequently underwent base-mediated intramolecular cyclization using KO*t*-Bu and air oxidation of the resulting 2,3-dihydro-4(1*H*)-quinolinone intermediates **7** in the presence of HCl to yield functionalized quinolin-4(1*H*)-one derivatives **6** in good to excellent yields.

**Scheme 5:**
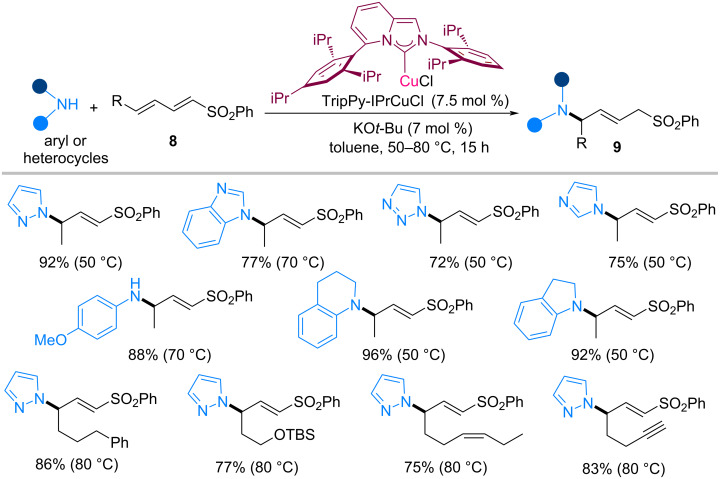
Copper-catalyzed 1,6-conjugate addition of aza-heterocycles to sulfonyl dienes.

Expanding beyond the classical 1,4-conjugate addition, Lee et al. reported the highly regio- and (*E*)-selective copper-catalyzed aza-1,6-conjugate addition of aza-heterocycles and arylamines to sulfonyl-1,3-dienes **8** (Scheme 5) [40]. The combination of the sterically demanding TripPy-IPrCuCl catalyst and KO*t*-Bu base proved crucial for controlling δ-selectivity over competing 1,4-addition pathways, affording (*E*)-allylic amines **9** in good to excellent yields with excellent regio- and stereoselectivity. The reaction exhibited high substrate tolerance toward various aza-heterocycles, including pyrazole, imidazole, and triazole derivatives. Notably, the sterically demanding and strongly electron-donating TripPy-IPr NHC ligand played a decisive role in directing the reaction pathway by stabilizing the copper–amido intermediate and promoting selective 1,6-conjugate addition, while suppressing the competing 1,4-addition pathways commonly observed in copper-catalyzed amination reactions.

**Scheme 6:**
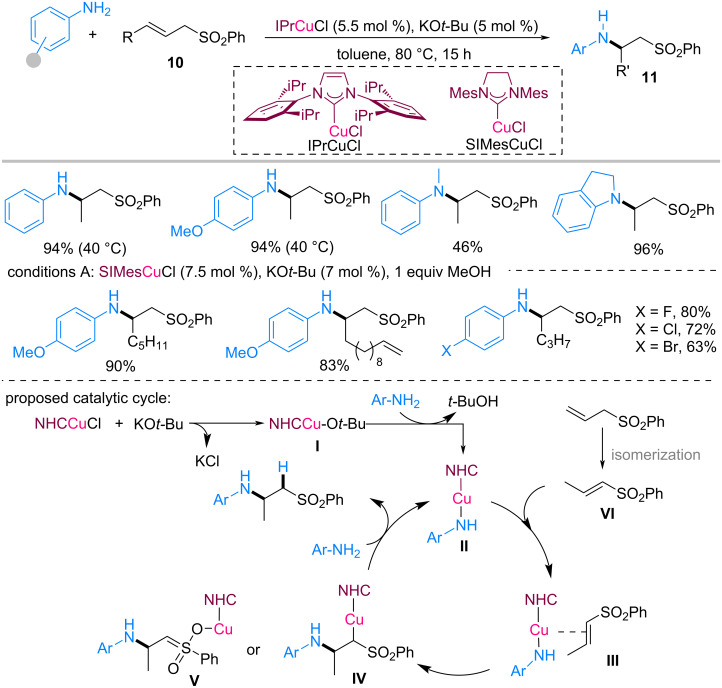
Cu-catalyzed aza-Michael addition of arylamines to allylic sulfones.

Recently, Lee et al. reported the copper-catalyzed hydroamination of allylic sulfones **10** (Scheme 6) [41]. By tuning the steric and electronic properties of the NHC ligands (e.g., IPrCuCl vs SIMesCuCl), highly β-regioselective amination reactions of arylamines with terminal and γ-substituted allylic sulfones **10** were achieved, affording diverse β-substituted β-amino sulfones **11** in high yields.

The reaction was proposed to proceed via a copper–amido catalytic cycle, analogous to the Gunnoe-type copper–amido aza-Michael addition mechanism. Initially, the NHC-CuO*t*-Bu complex **I**, generated from NHC–CuCl and KO*t*-Bu, underwent ligand exchange with arylamine, yielding the active NHC–Cu–amido species **II**. Allylic sulfone **10** was then isomerized by base or NHC–CuO*t*-Bu to vinyl sulfone **VI**, which coordinated to the copper–amido complex and underwent aminocupration or nucleophilic addition across the C=C bond, generating an organocopper intermediate **IV** or **V**. Subsequent protonation by aniline released the β-amino sulfone product and regenerated the copper–amido species, completing the catalytic cycle.

This mechanistic framework highlights the central role of the in situ-generated copper–amido complex and underscores the dual function of copper in substrate isomerization and C–N bond formation. This cooperative activation accounts for the high β-regioselectivity observed in Scheme 6 and further supports the copper–amido catalytic platform established by the Lee group.

**Scheme 7:**
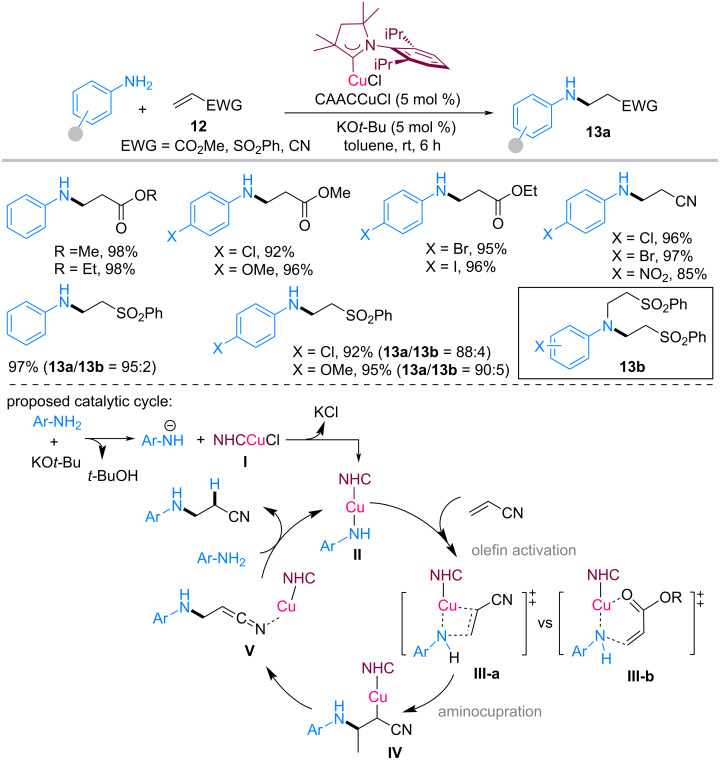
Cyclic(alkyl)(amino)carbene-copper-catalyzed aza-Michael addition.

Recently, Kundu et al. demonstrated that cyclic(alkyl)(amino)carbene (CAAC)-ligated copper complexes significantly enhanced the copper-catalyzed conjugate addition of arylamines to electron-deficient olefins [42]. As illustrated in Scheme 7, the use of an air- and moisture-stable (CAAC)Cu–Cl catalyst in the presence of KO*t*-Bu enabled the efficient hydroamination of activated olefins **12** bearing electron-withdrawing groups such as esters, nitriles, and sulfone substituents. Under these conditions, various anilines reacted efficiently to afford the corresponding β-amino carbonyl and β-amino sulfone products **13a** in excellent yields (85–98%). For the vinyl sulfone substrates, a small amount of dialkylated product **13b** was observed. Compared with earlier NHC-supported copper systems, the CAAC–Cu catalyst exhibited improved reactivity, particularly for ester-substituted alkenes. Mechanistic insights obtained from the DFT calculations suggested that the reaction proceeded via a copper–amido catalytic cycle involving metal-assisted olefin activation and the aminocupration pathway. In the initial step, deprotonation of the amine by KO*t*-Bu generated an amide species that coordinated to the copper center to form the catalytically active copper–amido intermediate **II**. Subsequent metal-assisted activation of the olefin led to the formation of C–N bond through aminocupration, generating organocopper intermediate **IV**. Computational studies indicated that this key step could occur through either four- or six-membered cyclic transition states (**III-a** vs **III-b**), depending on the nature of the electron-withdrawing group on the olefin. Finally, proton transfer from the amine substrate released the hydroamination product and regenerated the active Cu catalyst, completing the catalytic cycle.

#### Lewis acidic activation by Cu catalyst

**Scheme 8:**
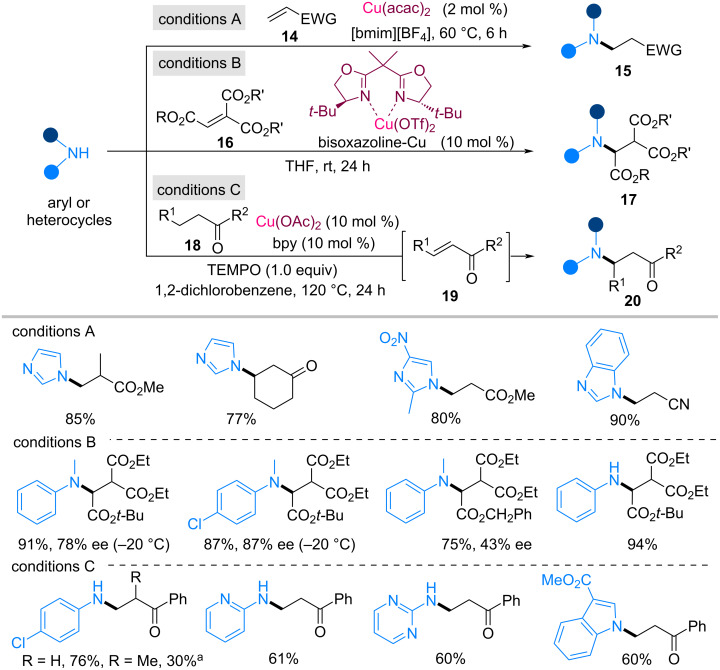
Aza-Michael addition enabled by Lewis acidic copper catalyst. ^a^20 mol % bpy, 50 mol % Na_2_CO_3_.

Scheme 8 shows three representative copper-catalyzed systems that highlight the versatility of copper species as Lewis acids in promoting aza-Michael reactions through distinct yet conceptually related activation modes. As reported by Kantam et al. (conditions A), Cu(acac)_2_ efficiently catalyzed the conjugate addition of imidazoles to α,β-unsaturated compounds **14** bearing an ester, ketone, or nitrile group [43]. In this transformation, copper functioned primarily as a Lewis acid by coordinating to the carbonyl group of the Michael acceptor. This coordination increased the electrophilicity of the β-carbon, facilitating nucleophilic attack. The reaction proceeded efficiently with various activated olefins **14**, affording *N*-substituted products **15** in high yields with good regioselectivities. Notably, the catalytic role of copper in this system was confined to electrophile activation, with the C–N bond formation proceeding via a classical Lewis acid-mediated aza-Michael pathway.

Yamazaki et al. reported that the conjugate addition of aromatic amines to highly activated ethenetricarboxylates **16** can be promoted by copper triflate species serving as Lewis acidic metal salts (Scheme 8, conditions B) [44]. Coordination of the Lewis acid to these electron-deficient alkenes substantially enhanced their reactivity toward nucleophilic attack, enabling the efficient formation of aza-Michael adducts **17** at ambient temperature. The authors also explored an asymmetric version of this reaction using chiral bisoxazoline–Cu(II) complexes. Coordination of ethenetricarboxylate substrate **16** to the chiral Lewis acid created a chiral environment that controlled the facial selectivity of the nucleophilic attack. The conjugate addition of *N*-methylaniline proceeded with moderate to good enantioselectivity, achieving 87% ee.

Mechanistically, the reaction follows a classical aza-Michael addition pathway, where the nitrogen lone pair of the aromatic amine attacks the β-carbon of the highly activated C=C bond, which is strongly polarized by the three ester substituents. Notably, the reaction outcome was highly dependent on the reaction conditions. Although mild conditions favored C–N bond formation, high temperatures promoted alternative pathways, such as aromatic substitution, indicating that the C–N bond formation was reversible under Lewis acidic conditions. Overall, this study highlights the key role of Lewis acid activation in controlling the reactivity and chemoselectivity of conjugate addition.

As reported by Su et al., copper catalysis can be further extended to a tandem dehydrogenation–conjugate addition sequence for the β-functionalization of saturated ketones (Scheme 8, conditions C) [45]. In this system, copper initially promoted oxidative dehydrogenation of ketone substrate **18** to generate an α,β-unsaturated intermediate **19** in situ via the radical-based pathway. The resulting enone **19** was subsequently activated through coordination to the copper center, facilitating nucleophilic addition of nitrogen sources to form β-amino ketones **20**. Although the initial desaturation step involved radical intermediates, the subsequent C–N bond formation proceeded via Lewis acid activation of the conjugated olefin. Therefore, copper played a dual role by generating and activating Michael acceptors within a single catalytic manifold.

**Scheme 9:**
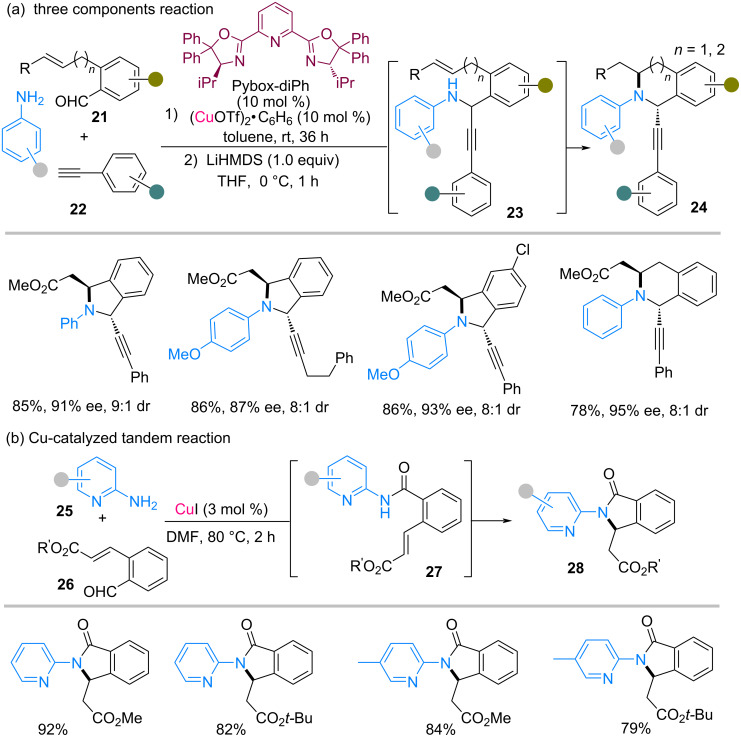
One-pot cyclization via aza-Michael addition enabled by Lewis acidic copper catalyst.

Building upon the simple Lewis acid-catalyzed aza-Michael additions described in Scheme 8, copper-catalysis has been successfully extended to cascade processes, where the initial conjugate addition is followed by intramolecular cyclization. Scheme 9 summarizes representative examples in which copper acts as a Lewis acid to promote the aza-Michael addition and subsequently enables annulation to construct diverse *N*-heterocycles.

As shown in Scheme 9a, Singh et al. reported that a Pybox-diPh–Cu catalytic system enables the one-pot imination-alkynylation-aza-Michael cascade for the enantio- and diastereoselective synthesis of 1,3-disubstituted isoindolines and tetrahydroisoquinolines **24** via a three-component coupling reaction involving 2-formylphenyl acrylates or 2-formylphenylcrotonates **21**, arylalkynes **22**, and anilines [46].

In this transformation, the chiral Pybox-diPh/Cu(OTf)_2_·C_6_H_6_ complex initially promoted imine formation followed by asymmetric alkynylation, generating the α,β-unsaturated ester intermediate **23**. Subsequent treatment with LiHMDS (lithium bis(trimethylsilyl)amide) induced base-promoted intramolecular aza-Michael cyclization, affording heterocyclic products **24**. Under optimized conditions, various substrates participated efficiently in the cascade process, providing the desired products in good yield with high enantioselectivity (up to 95% ee) and excellent diastereoselectivity.

Samanta et al. described a Cu(I)-catalyzed tandem oxidative amidation/aza-Michael reaction for the efficient synthesis of 3-substituted *N*-pyridinylisoindolinones **28** from 2-aminopyridines **25** and alkyl (*E*)-3-(2-formylphenyl)acrylates **26** (Scheme 9b) [47]. Under open-flask conditions at 80 °C in DMF, the reaction proceeded efficiently in the presence of CuI (3 mol %), affording the corresponding isoindolinone products **28** in good to excellent yields (79–92%). Mechanistically, the transformation involves a Cu(I)-promoted oxidative amidation followed by intramolecular aza-Michael cyclization. Initially, the condensation of amine with the aldehyde functionality generated a hemiaminal intermediate, which underwent Cu(I)-assisted air oxidation to produce the corresponding amide intermediate **27**. Subsequent intramolecular aza-Michael addition of the pyridinyl nitrogen to the activated α,β-unsaturated ester led to cyclization, ultimately furnishing the isoindolinone framework **28**. Mechanistic studies further revealed that electron-rich aminopyridines favor oxidative amidation, followed by intramolecular conjugate addition, whereas electron-deficient substrates might divert the reaction toward alternative cyclization processes.

**Scheme 10:**
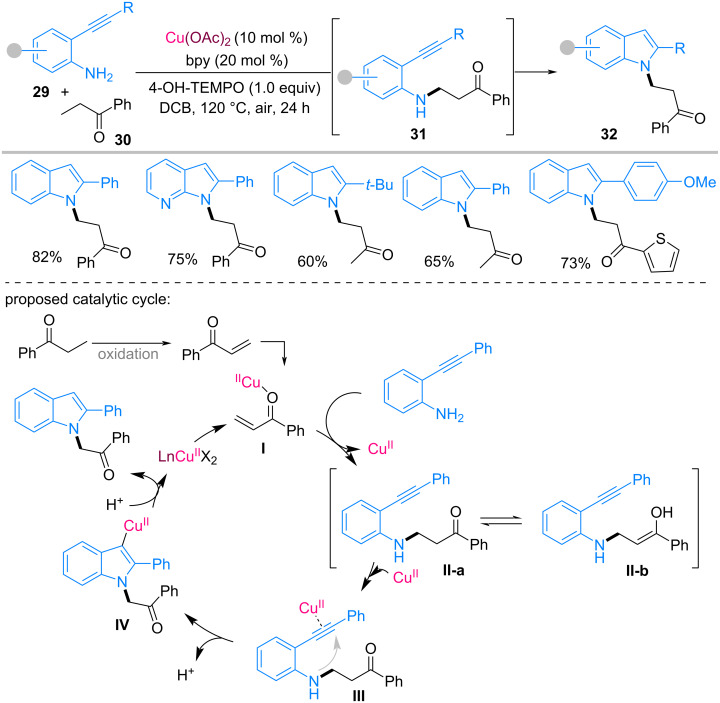
Lewis acidic Cu-catalyzed tandem reaction via aza-Michael addition.

Zhong et al. reported a copper-catalyzed cascade reaction for the synthesis of *N*-alkyl 2-arylindoles **32** from readily available 2-arylethynylanilines **29** and saturated ketones **30** under oxidizing conditions (Scheme 10) [48]. The transformation involved a sequential dehydrogenation, aza-Michael addition, and intramolecular annulation promoted by a Lewis acidic copper catalyst in the presence of Cu(OAc)_2_/bpy and 4-OH-TEMPO. Initially, the saturated ketone underwent copper-catalyzed oxidative dehydrogenation to generate the corresponding α,β-unsaturated ketone intermediate **I**. Activation of this enone by Cu(II) enabled aza-Michael addition of 2-arylethynylaniline **29** to form β-amino ketone intermediate **II**, which existed in equilibrium with its enol form. Subsequent coordination of the alkyne to the copper center promoted intramolecular 5-*endo-dig* hydroamination, yielding indole product **32** after proton transfer. Overall, the copper catalyst plays multiple roles, including the generation of Michael acceptor, activation of the electrophile, and facilitation of the annulation step, enabling the efficient construction of *N*-alkyl 2-arylindoles.

#### Heterogeneous processes: copper nanomaterials

**Scheme 11:**
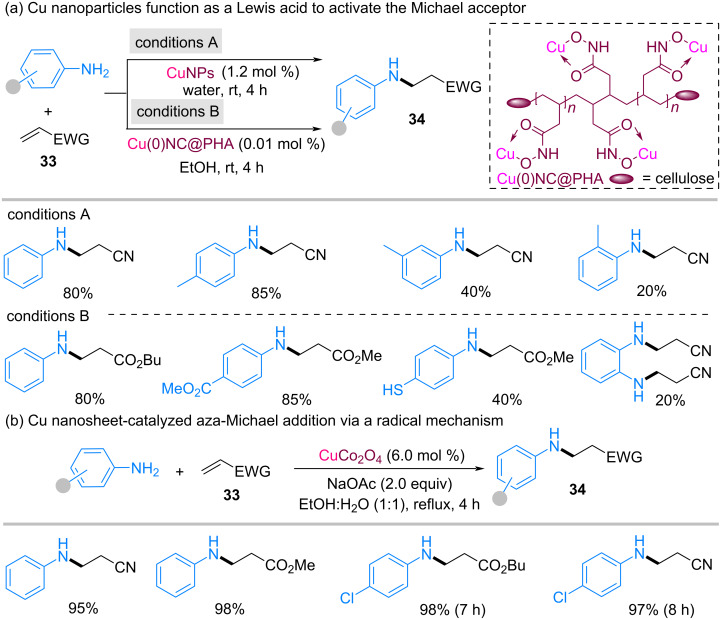
Copper nanomaterials-catalyzed aza-Michael addition via Lewis acid activation and radical pathways.

Scheme 11 summarizes the representative examples of heterogeneous copper nanomaterials used in aza-Michael addition reactions. In these systems, copper nanomaterials function as efficient heterogeneous catalysts that activate electron-deficient alkenes for the nucleophilic addition of amines under relatively mild conditions.

Rashid et al. reported that under conditions A, copper nanoparticles (CuNPs, 1.2 mol %) efficiently promoted the aza-Michael addition of anilines to activated alkenes at room temperature (Scheme 11a) [49]. In this system, the nanoparticulate copper surface acts as a Lewis acid to activate the Michael acceptor **33** through coordination to the electron-deficient C=C bond, which increases the electrophilicity at the β-position and facilitates nucleophilic attack by the amine. Consequently, various substituted anilines efficiently underwent conjugate addition to afford the corresponding β-amino products **34** in moderate to good yields under mild conditions. Furthermore, the heterogeneous catalyst could be readily recovered and reused without significant loss of activity, demonstrating the practical utility of NP-based copper catalysts for sustainable aza-Michael transformations.

Under conditions B, Rahman et al. developed a bio-supported copper nanocomplex, Cu(0)NC@PHA, derived from cellulose-based poly(hydroxamic acid) ligands [50]. The supported Cu(0) NPs catalyzed aza-Michael addition reactions of aromatic amines with various α,β-unsaturated esters **33** in ethanol at room temperature. The heterogeneous catalyst exhibited high efficiency, enabled easy recovery by filtration, and allowed recycling for up to five cycles without a significant loss of activity. Structural and spectroscopic analyses confirmed the successful immobilization of Cu NPs on the biodegradable support, and the catalytic activity was attributed to Lewis acid activation of the carbonyl group, which increased the susceptibility of the conjugated alkene to nucleophilic attack.

Scheme 11b highlights the study of Banerjee and Sharma, who developed sheet-like CuCo_2_O_4_ nanomaterials as efficient heterogeneous catalysts for aza-Michael addition reactions [51]. The CuCo_2_O_4_ catalyst was prepared via an oxalate decomposition method and extensively characterized using powder X-ray diffraction, field-emission scanning electron microscopy, and energy dispersive X-ray spectroscopy. Structural analysis revealed the formation of microsheet-like architectures with high crystallinity and well-defined lattice features, characteristic of the polycrystalline spinel phase of CuCo_2_O_4_. These sheet-like structures provide a large surface area and abundant exposed catalytic sites, which are advantageous for heterogeneous catalytic transformations.

Under optimized conditions using 6 mol % of CuCo_2_O_4_, various aromatic amines underwent conjugate addition to activated alkenes such as methyl acrylate, butyl acrylate, and acrylonitrile, affording β-amino carbonyl and nitrile products in good to excellent yields (95–98%). Control experiments demonstrated that the reaction was significantly inhibited in the presence of TEMPO under air atmosphere, supporting the involvement of a radical pathway. In contrast, high yields were obtained under inert conditions, which was consistent with radical stabilization during the catalytic process. Mechanistically, the CuCo_2_O_4_ surface enhanced β-carbon electrophilicity via copper-centered activation and radical generation, followed by the C–N bond formation and proton transfer, yielding the aza-Michael adduct.

Overall, these studies demonstrate that copper-based nanomaterials provide versatile heterogeneous platforms for aza-Michael reactions. Although CuNPs and supported copper nanocomplexes primarily operate through Lewis acid activation of the Michael acceptor, mixed-metal oxide nanomaterials, such as CuCo_2_O_4_, can promote alternative activation pathways involving radical intermediates.

### Copper-catalyzed hydroamination of alkenes

The hydroamination of alkenes is one of the most atom-economical strategies for C–N bond formation because it enables the direct addition of an N–H bond across a C=C double bond without requiring prefunctionalized substrates. Despite their conceptual simplicity, hydroamination reactions often suffer from limited reactivity and selectivity, particularly when aromatic amines are used as nucleophiles. Recent advances in copper catalysis have revealed that hydroamination can proceed through several distinct mechanistic pathways, including copper–amido mediated amination, Lewis acid-catalyzed processes, and radical-mediated processes.

#### Hydroamination via copper–amido intermediates

Gunnoe et al. reported the copper-catalyzed hydroamination involving copper–amido intermediates [52]. As discussed in the previous section, Gunnoe et al. developed a well-defined NHC-based copper–amido complex, IPrCu–NHPh, which efficiently catalyzed the aza-Michael addition of aniline to activated olefins [32–34]. Notably, the same catalytic platform promoted the hydroamination of vinylarenes, providing an early demonstration of copper-catalyzed hydroamination with aromatic amines.

**Scheme 12:**
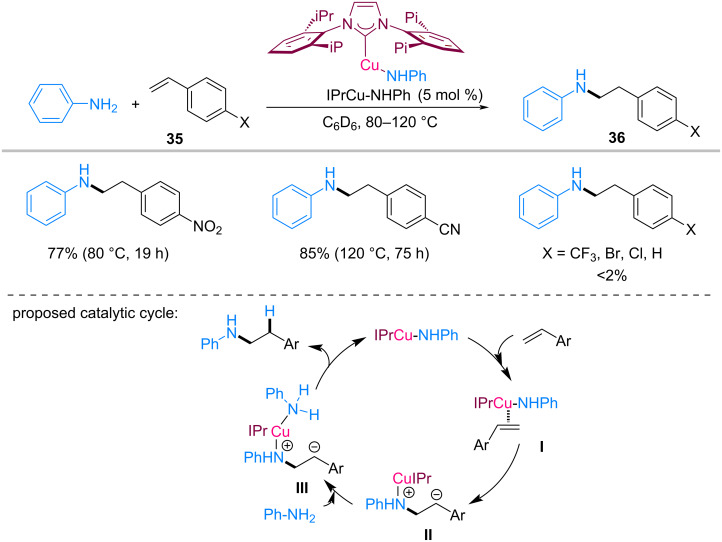
Copper–amido-mediated hydroamination of aniline with vinylarenes.

In their study, well-defined IPrCu–NHPh catalyzes the hydroamination of vinylarenes under relatively simple conditions (Scheme 12). In the presence of 5 mol % IPrCu–NHPh in C_6_D_6_, aniline was reacted with styrene derivatives bearing strong electron-withdrawing substituents to yield the corresponding anti-Markovnikov hydroamination products. For example, *p*-nitrostyrene and *p*-cyanostyrene afforded the desired β-amino products in 77% and 85% yield, respectively. In contrast, vinylarenes containing less electron-withdrawing substituents (X = CF_3_, Br, Cl, or H) exhibited minimal or no reactivity under identical conditions, highlighting the critical role of alkene electrophilicity in facilitating C–N bond formation.

Mechanistic studies suggested that the reaction proceeded via the direct nucleophilic addition of the copper–amido ligand to vinylarene. As illustrated in the proposed catalytic cycle, the copper–amido complex initially coordinated to the electron-deficient vinylarene. The amido ligand then underwent regioselective intermolecular nucleophilic attack at the less substituted β-carbon of the alkene, generating zwitterionic intermediate **II**. Subsequent proton transfer from aniline released the anti-Markovnikov β-amino product **36** and regenerated the copper–amido catalyst.

The strong dependence of the reactivity on the electron-withdrawing substituent of the vinylarene was consistent with the rate-determining nucleophilic addition step. Although the substrate scope in this early study is relatively limited, these results provide critical mechanistic insights into copper-catalyzed hydroamination and demonstrate that copper–amido complexes can directly participate in alkene amination via nucleophilic addition pathways.

**Scheme 13:**
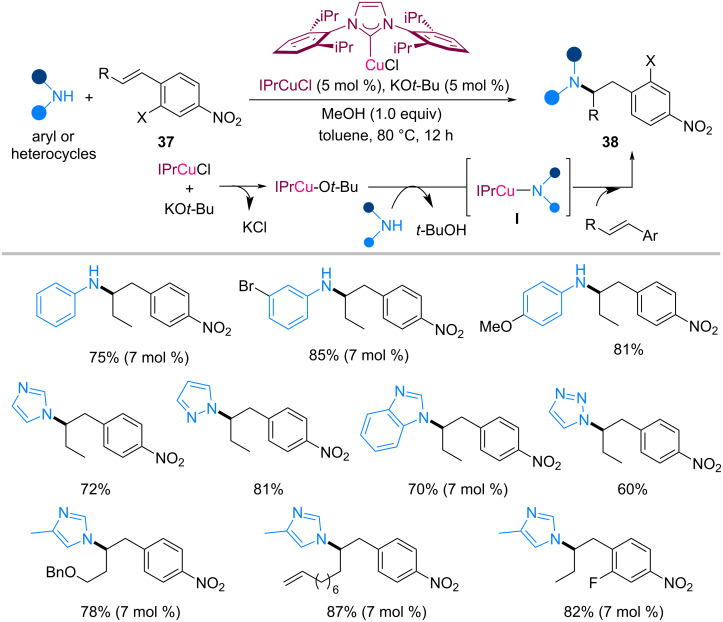
Copper-catalyzed anti-Markovnikov hydroamination of (hetero)aromatic N–H with vinylarenes.

Given that copper–amido intermediates can participate in alkene amination, further developments have expanded the scope of copper-catalyzed hydroamination. For example, Lee et al. reported the intermolecular hydroamination of vinylarenes with arylamines and aza-heterocycles using a readily accessible NHC–copper catalyst system (Scheme 13) [53]. The reaction used IPrCuCl (5 mol %) in combination with KO*t*-Bu as a base and MeOH as an additive in toluene at 80 °C, enabling the direct addition of various nitrogen nucleophiles to nitrostyrene derivatives **37** with excellent anti-Markovnikov selectivity. Under the optimized conditions, various arylamines and *N*-heterocycles, including imidazoles, benzimidazoles, pyrazoles, and triazoles, underwent efficient hydroamination to afford the corresponding β-amino products **38** in good to excellent yields. Notably, sterically demanding or functionalized substrates were well tolerated, and the catalytic system enabled the hydroamination of unsymmetrical 1,2-disubstituted vinylarenes **37**, which were typically challenging substrates for intermolecular hydroamination reactions. These results demonstrate that the Cu/NHC catalytic system significantly expands the substrate scope compared to earlier copper-amido systems. This transformation is proposed to proceed through the in situ generation of NHC-copper–amido species **I**, formed via the reaction of an IPrCu–O*t*-Bu intermediate with an amine substrate.

**Scheme 14:**
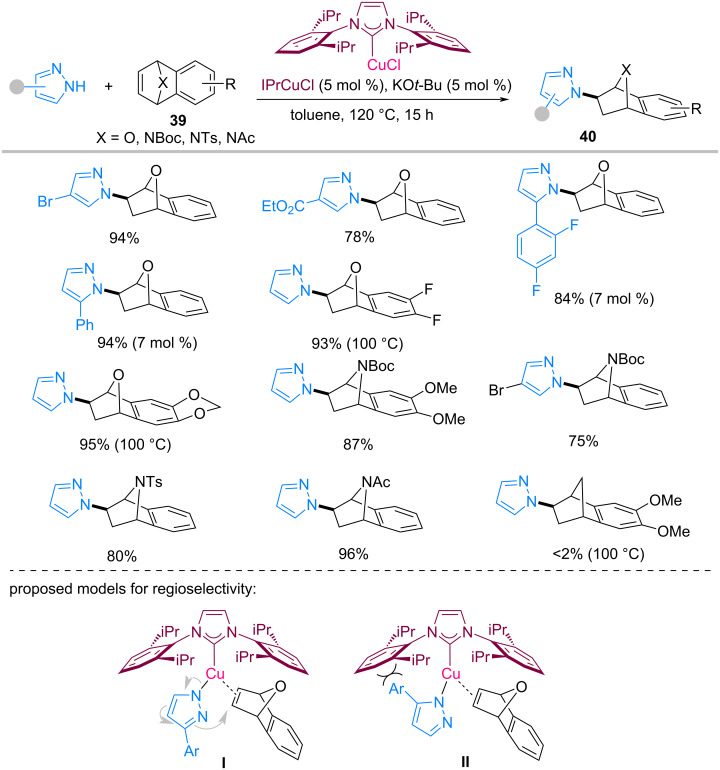
Hydroamination of oxa- and azabenzonorbornadienes with pyrazoles.

The concept of copper-catalyzed hydroamination via copper–amido intermediates has also been extended to strained bicyclic alkenes. Lee and Kim reported the NHC–copper-catalyzed hydroamination of oxa- and azabenzonorbornadienes **39** with pyrazoles (Scheme 14) [54]. Using IPrCuCl (5 mol %) in combination with KO*t*-Bu in toluene, various pyrazole derivatives efficiently reacted with bicyclic alkenes **39** to afford the corresponding exoselective hydroamination products **40** in good to excellent yields. The reaction exhibited high functional group tolerance, including that for halogen-, ester-, and aryl-substituted pyrazoles, and provided hydroaminated bicyclic product **40** with high chemo- and stereoselectivity. In addition, oxa- and azabenzonorbornadiene substrates bearing diverse substituents on the aromatic ring were compatible under the optimized conditions, yielding the desired products in up to 96% yield.

**Scheme 15:**
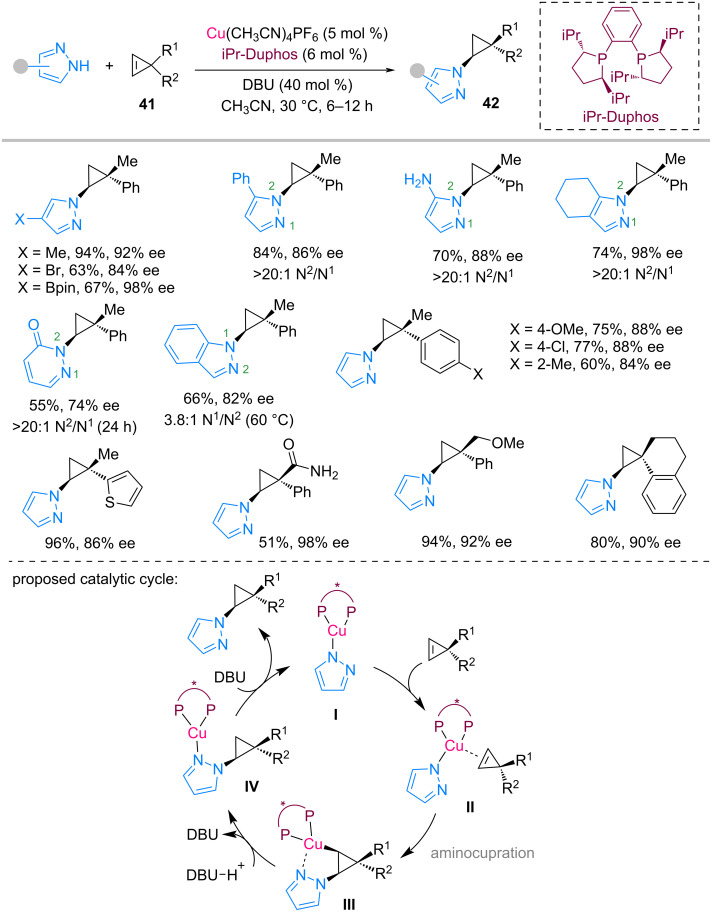
Copper-catalyzed enantioselective hydroamination of cyclopropenes with pyrazoles.

A plausible mechanism involves the in situ generation of an NHC-ligated copper–amido species via ligand exchange between the NHC–CuO*t*-Bu complex and pyrazole substrate (Scheme 15). Subsequent stereoselective aminocupration of the strained bicyclic alkene, followed by proton transfer from the pyrazole, regenerated the copper–amido species and released the hydroamination product. The observed exo-selectivity and regioselectivity with unsymmetrical pyrazoles were attributed to steric interactions between the substituent on the pyrazole ring and the bulky 2,6-diisopropylphenyl groups of the IPr ligand, which preferentially stabilized the specific orientation of the copper–pyrazolyl intermediate (**I** vs **II**).

Recently, Dong et al. reported the enantioselective hydroamination of cyclopropenes with pyrazoles using a copper catalyst, affording chiral cyclopropyl pyrazoles with high levels of regio-, diastereo-, and enantioselectivities (Scheme 15) [55]. In the presence of Cu(CH_3_CN)_4_PF_6_ (5 mol %) and the chiral bisphosphine ligand (*R*,*R*)-iPr-DuPhos, the coupling of pyrazoles with substituted cyclopropenes proceeded efficiently at 30 °C in CH_3_CN with DBU as a base. Under these conditions, various pyrazoles underwent hydroamination to furnish the corresponding *N*-cyclopropylpyrazoles in moderate-to-excellent yields with excellent stereocontrol, typically yielding >20:1 diastereomeric ratios and up to 98% ee. The reaction exhibited a broad substrate scope with respect to the pyrazole and cyclopropene components. Symmetrical pyrazoles bearing alkyl, halogen, or electron-withdrawing substituents afforded the desired products in 40–94% yield with excellent enantioselectivity. Unsymmetrical pyrazoles also reacted efficiently, exhibiting remarkable nitrogen regioselectivity and favoring a more sterically hindered N^2^ position (N^2^/N^1^ > 20:1). Furthermore, various substituted cyclopropenes were well tolerated, yielding the corresponding hydroamination products in good yield with high enantioselectivity. Mechanistic studies suggested that the reaction proceeded via a copper–pyrazolate (copper–amido) catalytic cycle [56]. The active copper–amido species **I** initially coordinated to the cyclopropene to form a π-complex **II**, followed by cis-aminocupration across the strained alkene, generating a cyclopropylcopper intermediate **III**. Subsequent protodemetalation released the hydroamination product and regenerated the Cu catalyst. Experimental and computational studies further supported the five-center aminocupration transition state, which accounted for the unusual N^2^-selectivity of pyrazole nucleophiles. This study presents the first asymmetric hydroamination of cyclopropenes involving heterocyclic amines via the copper–amido mechanism.

#### Hydroamination via Brønsted acidic or Lewis acidic activation by Cu

**Scheme 16:**
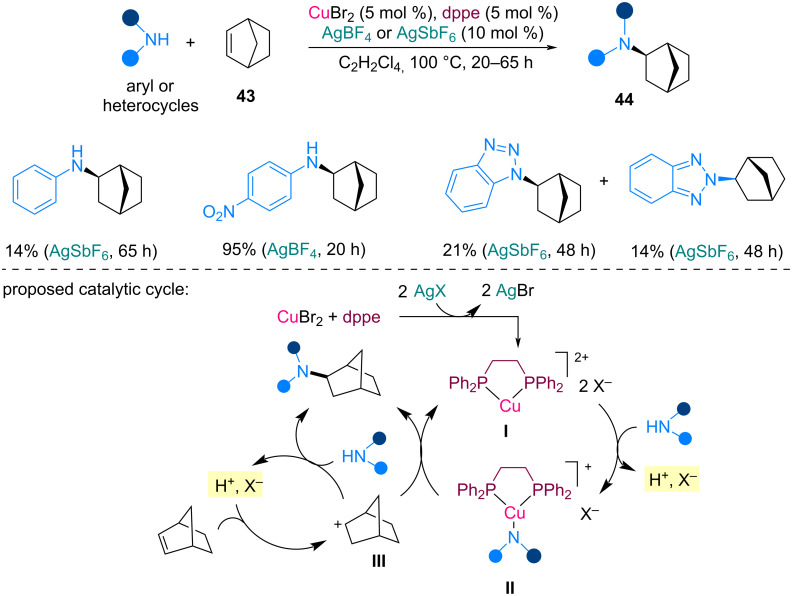
Copper/Brønsted acid system for hydroamination of strained alkenes.

An alternative strategy for copper-catalyzed hydroamination of unactivated alkenes was reported by Agbossou-Niedercorn et al., who utilized a catalytic system comprising CuBr_2_, a phosphine ligand (dppe), and a silver salt (Scheme 16) [57]. Under these conditions, various aryl- or heteroarylamines underwent intermolecular hydroamination with strained or unactivated alkenes **43** such as norbornene, to give the corresponding amination products **44**. However, in some cases, only moderate yields were obtained. For example, aniline provided the hydroamination product in 14% yield, whereas more electron-deficient amines, such as *p*-nitroaniline, afforded significantly improved yields of up to 95%. Mechanistic investigations revealed that this reaction proceeded through a pathway distinct from the classical metal–amido mediated hydroamination. The combination of copper halide and silver salt generated a cationic copper complex **I**, which, upon coordination of the amine substrate, led to proton release and formation of a Brønsted acid species in situ. Control experiments demonstrated that the addition of bases suppressed the reaction, whereas the reaction proceeded efficiently in the presence of catalytic amounts of HBF_4_, indicating that the hydroamination was predominantly promoted by acid catalysis rather than by a discrete copper–amido insertion pathway. Accordingly, the proposed catalytic cycle involved protonation of the alkene to generate carbocationic intermediate **III**, followed by nucleophilic attack of the (hetero)aromatic amine or copper–amido intermediate **II** to furnish the hydroamination product. In this system, the copper complex acts as a precatalyst for the generation of active Brønsted acid, highlighting that copper-based hydroamination reactions can proceed through mechanistically distinct pathways depending on the catalyst system used.

**Scheme 17:**
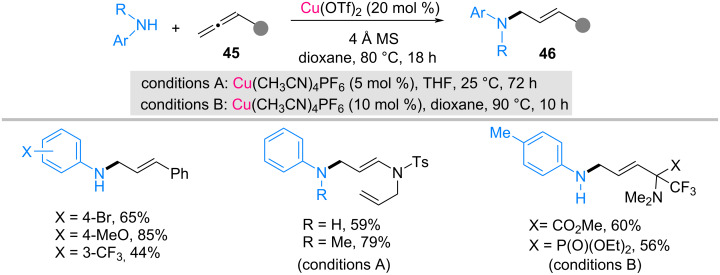
Hydroamination of allenes enabled by Lewis acidic copper catalyst.

Monnier et al. reported a copper-catalyzed intermolecular hydroamination of allenes for the regio- and stereoselective synthesis of (*E*)-allylamines **46** from terminal allenes **45** and aniline derivatives (Scheme 17) [58–60]. In the presence of Cu(OTf)_2_ or Cu(CH_3_CN)_4_PF_6_, various monosubstituted allenes bearing aryl, aminocarboxylate, aminophosphonate, and sulfonamide groups smoothly reacted with aniline derivatives or *N*-methylaniline to afford the corresponding linear (*E*)-allylamine products **46** with excellent regio- and stereoselectivity. The nucleophilic amine selectively added to the terminal carbon of the allene, providing exclusively the formal anti-Markovnikov hydroamination products. Mechanistic studies suggested that the catalytically active species is a cationic Cu(I) complex, generated in situ from the Cu(II) precursor, which activates the allene toward nucleophilic attack by the amine and controls the high regio- and stereoselectivity of the transformation [61].

#### Hydroamination via radical pathways

**Scheme 18:**
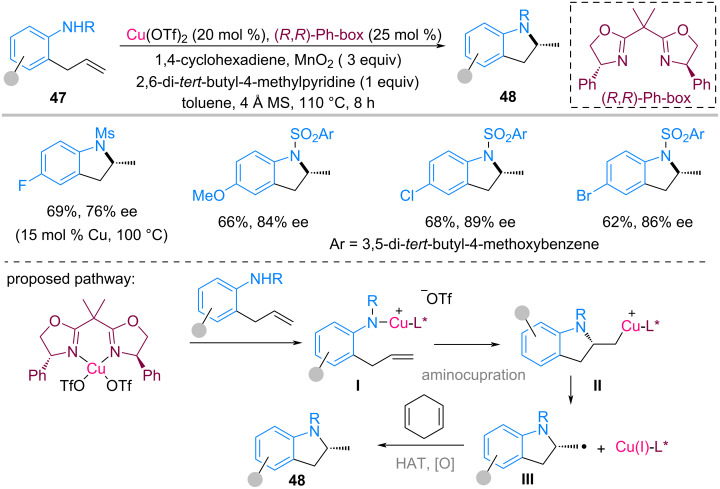
Enantioselective intramolecular hydroamination through aminocupration followed by radical processes.

Chemler et al. reported a significant advancement in Cu-catalyzed hydroamination by performing the enantioselective intramolecular hydroamination of *N*-sulfonyl-2-allylanilines **47** to access chiral 2-methylindolines **48** (Scheme 18) [62]. Using a catalytic system composed of Cu(OTf)_2_ and the chiral bis(oxazoline) ligand (*R*,*R*)-Ph-box, the cyclization of *N*-sulfonyl-2-allylaniline derivatives **47** proceeded efficiently in the presence of 1,4-cyclohexadiene as a hydrogen atom donor and MnO_2_ as a stoichiometric oxidant. Various substituted substrates underwent intramolecular hydroamination to furnish the enantioenriched indoline products **48** in moderate-to-good yields, with up to 89% ee. Mechanistic studies have suggested that the reaction proceeds via the *cis*-aminocupration pathway, in which a nitrogen nucleophile and copper(II) are added across the alkene to form the organocopper intermediate **II** [63]. This intermediate is proposed to undergo homolytic cleavage to generate carbon-centered radical **III** and a Cu(I) species, following which hydrogen atom transfer from 1,4-cyclohexadiene provides the hydroamination product and regenerates the catalytic copper species. This strategy represents one of the earliest examples of enantioselective copper-catalyzed alkene hydroamination, and highlights a mechanistically distinct pathway involving radical intermediates generated from aminocupration, in contrast to previously discussed nucleophilic copper–amido addition or Brønsted acid-mediated processes.

**Scheme 19:**
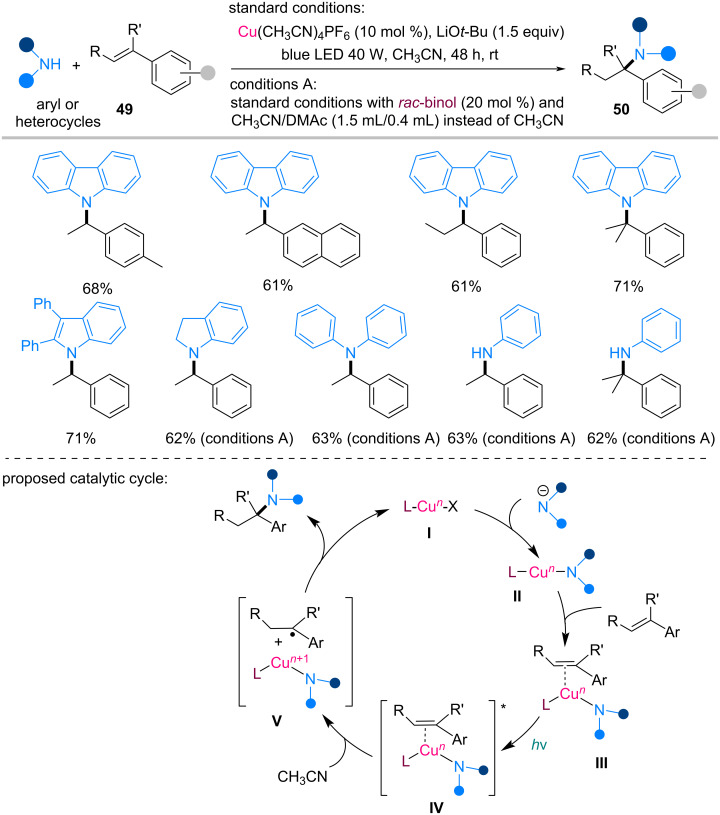
Visible-light-induced copper-catalyzed hydroamination of styrenes.

Recent studies have demonstrated that copper catalysts promote hydroamination via photoinduced radical pathways. In 2019, Zhang and Xiong reported the visible light-induced intermolecular hydroamination of alkenes using simple aryl- and heteroarylamines (Scheme 19) [64]. Cu(CH_3_CN)_4_PF_6_ (10 mol %) and LiO*t*-Bu in CH_3_CN under blue LED irradiation enabled the coupling of alkenes **49** with carbazoles, indoles, and aniline derivatives to afford the corresponding amination products **50** with Markovnikov regioselectivity. Various styrene derivatives bearing electron-donating or electron-withdrawing substituents were compatible with the reaction, affording the desired amines **50** in moderate to good yields. Mechanistic experiments indicated that the reaction proceeded via a photoexcited copper complex and radical intermediates. In the presence of a base, ligand exchange between copper precursor **I** and the amine generated the copper–amido species **II**. This complex subsequently coordinated with the alkene to form the copper–amine–alkene complex **III**. Upon visible-light irradiation, complex **III** was photoexcited to yield the excited copper complex **IV**. Subsequently, the excited complex **IV** underwent a single-electron transfer process accompanied by hydrogen atom abstraction from CH_3_CN, generating a benzyl radical and a higher-valent organocopper intermediate **V**. The benzyl radical was subsequently captured by the copper species to furnish the hydroamination product while regenerating the copper catalyst. Radical trapping experiments and deuterium labeling studies supported the involvement of radical intermediates and solvent-derived hydrogen atom transfer steps in the catalytic cycle. This study highlights the dual role of copper as a photocatalyst and coupling catalyst, enabling hydroamination under mild and operationally simple conditions.

**Scheme 20:**
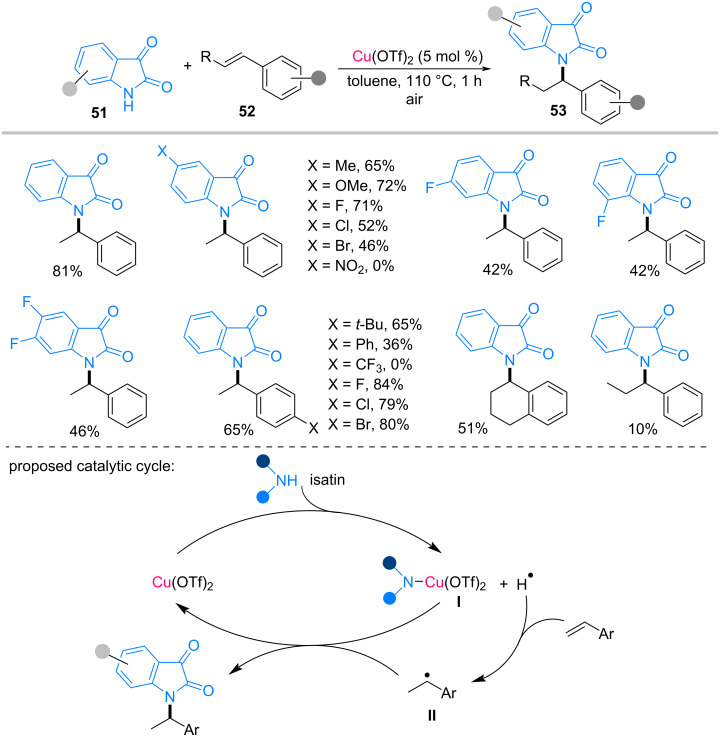
Lewis acid-catalyzed radical hydroamination of styrenes with isatins.

Recently, Qiu et al. reported a Lewis acid-mediated hydroamination of styrenes with isatin derivatives catalyzed by Cu(OTf)_2_ under air atmosphere (Scheme 20) [65]. Under optimized conditions (5 mol % Cu(OTf)_2_ in toluene at 110 °C), various isatins **51** reacted smoothly with styrene derivatives **52** to afford the corresponding Markovnikov hydroamination products **53** in moderate to good yields. Mechanistic investigations have suggested that the transformation proceeded via a radical pathway rather than a classical ionic hydroamination process. Deuterium-labeling experiments revealed that the hydrogen incorporated into the product originated from the N–H bonds of isatin. Furthermore, the addition of a radical scavenger, such as TEMPO, completely inhibited the reaction. Moreover, radical clock experiments with cyclopropyl-substituted styrenes resulted in ring-opened products, providing strong evidence for the involvement of alkyl radical intermediates. Based on these observations, we proposed a plausible reaction mechanism (Scheme 20). Initially, Cu(OTf)_2_ activated the N–H bond of isatin to generate copper complex **I** and a hydrogen radical. The hydrogen radical subsequently added to styrene to form benzylic radical intermediate **II**, which preferentially yielded Markovnikov product **53**. Finally, the recombination of this radical intermediate with the copper-nitrogen species **I** furnished the hydroamination product while regenerating the copper catalyst. This study demonstrates that simple Lewis acidic copper salts can promote hydroamination through radical pathways, offering a complementary strategy to previously reported copper–amido nucleophilic addition or aminocupration mechanisms.

## Conclusion

Copper catalysis has emerged as an efficient and versatile platform for the direct hydroamination of alkenes with (hetero)aromatic N–H nucleophiles. The success of these transformations arises from the diverse roles that copper plays in alkene amination. In particular, copper catalysts can promote C–N bond formation via copper–amido-mediated nucleophilic addition, copper–amido-mediated aminocupration, Lewis acid–type alkene activation, and radical pathways. These complementary activation modes enable hydroamination across a variety of alkene classes while providing control over the regioselectivity and functional group tolerance. Advances in ligand design and catalytic system development have further expanded the scope and efficiency of copper-catalyzed hydroamination. Despite these achievements, extending these methods to electron-neutral and sterically hindered unactivated alkenes, such as simple aliphatic terminal alkenes and internal alkenes, as well as achieving higher levels of stereocontrol, remains challenging. Continued catalyst development and mechanistic insight will further advance the synthetic utility of copper-catalyzed direct hydroamination reactions.

## Data Availability

Data sharing is not applicable as no new data was generated or analyzed in this study.
